# PPARs and Gastrointestinal Cancer

**DOI:** 10.1155/2012/918079

**Published:** 2012-11-14

**Authors:** Valerio Pazienza

**Affiliations:** Research Laboratory of Gastroenterology Unit, IRCCS “Casa Sollievo della Sofferenza” Hospital, 71013 San Giovanni Rotondo, Italy

Since the discovery of the peroxisome proliferator-activated receptors (PPARs) in *Xenopus* frogs as receptors that induce the proliferation of peroxisomes in cells, the study of these factors has received widespread interest. After the characterization of the different isoforms (PPAR*α*, PPAR*β*/*δ*, and PPAR*γ*) of these nuclear receptors in the 90s, numerous ligands have been described. Although the responsibility of PPARs in cancer development and progression is still controversial, many scientists consider PPARs as potential biomarkers/targets for cancer prevention and therapy. 

Numerous mechanisms of PPARs action have been well reported; however the behaviour and function of PPARs differs within the context of different types of tissues and also depends on etiologic agents which could be one of the reasons to explain the conflicting literature.

The aim of this SI was to assemble different studies in order to better establish the functions of PPARs in the context of different types of gastrointestinal cancer (GI). 

There are two research papers published by our group and nine reviews covering the main aspects of all types of GI cancer with the aim of elucidating the role of PPARs by confining their function to the different organs taken in consideration by the different authors.

In our research article entitled “*Time-qualified patterns of variation of PPAR*γ*, DNMT1, and DNMT3B expression in pancreatic cancer cell lines,*” we assessed the time-related patterns of variation of PPAR*γ* and DNMTs in pancreatic cancer (PC) cell lines after synchronization in order to understand the circadian behaviour of these factors. Our data demonstrated the temporal influence (over 24 h) on PPAR*γ* expression in PC cells. The circadian fluctuation of PPAR*γ* expression is an important finding that could help to understand the many disagreeing studies. A temporal variation of PPAR*γ* mRNA expression over the day has to be taken into account when performing an “*in vitro*” or “*in vivo*” study.

In our study “*Correlations among PPAR*γ*, DNMT1, and DNMT3B expression levels and pancreatic cancer*,” we sought to investigate the relationship among PPAR*γ* and the DNA-methyltransferases in PC patients and in “*in vitro*” models of PC cell lines to better understand the role of PPAR in epigenetic modification. We demonstrated that PPAR*γ* expression is positively associated with DNMT1 but not with DNMT3B whose higher expression, however, was significantly associated to a lower mortality in a cohort of PC patients.

A. Stravodimou et al. described the regulation of these nuclear receptors by the ubiquitin-proteasome system in pancreatic cancer suggesting that inhibition of specific ubiquitination enzymes (instead of the proteasome) could also be a solution for a more specific pharmacologic interventions. 

In the review article “*The role of peroxisome proliferator-activated receptors in the esophageal, gastric, and colorectal cancer*” by A. Fucci et al., the authors stressed out the promising translational outcome of the reported studies on nuclear receptors, raising the possibility of identifying PPAR alterations in premalignant lesions so that they can be used as prognostic biomarkers, whilst J.-I. Park and J.-Y. Kwak, in the review “*The role of peroxisome proliferator-activated receptors in colorectal cancer*,” highlight the role of PPARs as target for cancer therapy. An interesting message appears in “PPAR*γ* in inflammatory bowel disease” by V. Annese et al. After describing the potential of PPAR*γ* in inflammatory-induced mechanisms, the authors reflected on the link between microbiota and PPAR*γ* receptor, considering it worth further studies, since some commensal bacterial or natural ligands of foods may directly activate and increase the expression of PPAR*γ*, thus determining a “biologic” anti-inflammatory action.

A paper by J.-M. Lee et al. about “*The role of PPAR*γ* in Helicobacter pylori infection and gastric carcinogenesis*” reported that in gastric epithelial cells of *H. pylori*-infected patients a strong nuclear staining was present as compared to *H. pylori*-negative subjects.

As for the liver, a reconciling model based on mitochondria-related features to resolve the conflicting results on PPARs-Liver-HCV is offered by F. Agriesti et al. in “*PPARs and HCV-related hepatocarcinoma: a mitochondrial point of view*.” 

Conversely the following two reviews indicate possible future research directions. M. Peyrou et al., in “*PPARs in liver diseases and cancer: epigenetic regulation by microRNAs*” dealt with the epigenetic regulation of PPARs expression and activity by miRNAs considering this new field of research to still be in its infancy and suggesting that alterations of the expression/activity of PPARs isoforms by distinct miRNAs could represent critical molecular mechanisms involved in the physiopathology of each organ undergoing a PPAR-dependent control; alternatively G. P. Ables, in “*Update on Ppar*γ* and nonalcoholic fatty liver disease*,” invites the investigators to elucidate the effect of specific conformational and structural differences between the nuclear receptor and its ligands concluding that the advent of the development of SPPARMS moves in the direction of specifically eliciting the desirable effects of PPAR*γ* activation.

In conclusion, even though PPARs could have prognostic and/or therapeutic roles, there is an urgent need to shed light on the favorable potential or harmful risk of their modulator. Taking into account all the contributions made by the authors to this SI, our hope is that a step forward is made to resolve, at least partially, the conflicting data existing in the literature and to give interesting future research directions for a wide range of researchers.

Finally, I wish to thank the two guest coeditors, Dr. Manlio Vinciguerra and Dr. Gianluigi Mazzoccoli, for their work on this SI, and a warm thank you to the authors who have contributed to this special issue dedicating our effort to all those individuals who lead a brave daily battle against cancer.

*Valerio Pazienza* *Valerio Pazienza*

